# Anthropometrical measurements of external ear between normal and diabetic patients

**DOI:** 10.6026/973206300222259

**Published:** 2026-04-30

**Authors:** Jaya Deshmukh, Harsh Kumar Chawre, Pawan Kumar Sharma

**Affiliations:** 1 Department of Anatomy, Gandhi Medical College, Bhopal, Madhya Pradesh, India; 2 Department of Anatomy, GMC DATIA, Madhya Pradesh, India; 3 Department of Otolaryngology, Dr. Laxminarayan Pandey Government Medical College, Ratlam, Madhya Pradesh, India

**Keywords:** Human ear, diabetes, anthropometric measurements

## Abstract

Although external ear anthropometry is a cornerstone of forensic and reconstructive science, the morphological impact of systemic metabolic disorders remains unexplored. Therefore, it is of interest to compare bilateral external ear measurements between diabetic and healthy individuals to determine if chronic hyperglycemia significantly influences soft tissue morphology. Anthropometric data were collected from both cohorts and categorized by sex to evaluate symmetry and quantify deviations in ear dimensions. Thus, we show that right ear measurements were consistently larger than those of the left ear in both groups, with diabetic patients exhibiting statistically significant differences in specific morphological parameters compared to the control group. In each group, the measurements of the right ear for men and women were larger than those of the left ear, and there were significant differences in anthropometric measurements.

## Background:

The external ear refers to the outermost part of the ear which is responsible for collecting sound and directing it into the auditory system [1]. It is made up of two primary components: the auricle and the external auditory canal [2]. The ears influence the appearance of individuals and contribute to the aesthetics of the human face, as the external ear consists of the auricle (or pinna) and the external auditory meatus [3]. The auricle is usually associated with congenital anomalies as a malposition related to Down's and Turner's syndrome with injuries and pathological conditions as in cancer, the auricle has often acquired defects [4]. Correcting these conditions needed information on the usual auricular measurements because recent anthropometric measurement studies show a lot of variability depending on age, sex and ethnicity and there is a difference between right and left ears even in the same person [5]. The perception of human differences has an important role in plastic surgery, prosthetics [6]. In addition to the fact that both the length and the width of the ear increase with age [3]. Therefore, it is of interest to compare the anthropometric measurement of the external ear of right and left ears in normal persons and diabetic patients.

## Methodology:

This cross-sectional study was conducted at an outpatient clinic in Bhopal after prior consent was obtained from the participants and ethical permission was granted. Data were collected from November, 19 2024, to February 20 2025. Two groups were divided based on the presence or absence of diabetes. Individuals with Glycated hemoglobin (Hb1Ac) level of > 6.5% were considered diabetic. All subjects with congenital auricular malformation of the external ear (pinna) and all individuals with a history of trauma to the external ear (pinna) or surgery were not included in the study. Anthropometric tests for the external ear were performed, including total ear length (TEL), total ear width (TEW), total lobular length (TLL), and total lobular width (TLW), which were calculated by a specific investigator using a Vernier caliper. The questionnaire framework was formulated with the help of experts for the purpose of our analysis, following the guidance received from the institutional ethics committee. Data were collected after obtaining verbal consent from the patients. The anthropometric measurements of the external ear, specifically the "pinna," included the total ear length (TEL), which measures the distance from the highest point of the helix to the lowest point of the ear lobule. In a similar manner, the total width of the ear (TEW) measures the distance between the posterior point and the most anterior point of the auricle. The total length of the lobule (TLL) is calculated as the distance from the bottom of the lobule to the base of the tragus notch. The total lobule width, measured at the midpoint of the lobular height, represents the horizontal width of the lobule. For each subject, the measurements were performed twice to ascertain accuracy, and the arithmetic mean of the two measurements was used for each dimension. Numerical data were analyzed using JAMOVI. Comparisons of measurements according to sex were performed using an independent samples t-test. Comparisons of measurements obtained from the right and left ears of a given sex were performed using a paired sample t-test.

## Results:

The sample size was 190 cases separately classified into two categories with 95 normal persons and 95 type II mellitus patients. The sex wise distribution was females 55(58%) and males 40(42%) in each group. The patient's age ranged from 40 to 60 years (50±9.8) in both the groups. The anthropometric measurements of right ear in male diabetes mellitus patients (TEL, TEW, TLL and TLW) respectively were 6.65±0.61, 2.93±0.28, 2.01±0.30, 1.97 ±0.25 but in the left ear they were 6.42 ±0.44, 2.89±0.49, 1.90±0.31, 1.94±0.18 while in the female diabetes mellitus patients; the right ear (TEL, TEW, TLL and TLW) respectively were 6.10±0.39, 2.78±0.25, 1.72±0.21, 1.75±0.16 but in the left ear are 6.12±0.45, 2.37±0.26, 1.70±0.23, 1.57±0.17 as shown in Table 1 (see PDF). Here still the measurements are larger in males than females and the right auricle measurement is larger than left in both female and male patients. TEL (Total Ear Length): was shorter in diabetics and the difference was not statistically significant. TEW (Total Ear Width) was significantly narrower in diabetics. There were no significant differences noted with respect to TLL (Total Lobe Length) and TLW (Total Lobe Width).

## Discussion:

The ear is a significant but often overlooked feature of the face, with its shape and size providing insights into an individual's age and sex. The main role of the pinna is to gather sound waves that travel to the eardrum via the external auditory canal; the ear is also acknowledged as a cosmetic feature, with its significance being more closely tied to the aesthetics and physiognomy of the face [9]. Our research indicates that all anthropometric measurements in males exceed those in females, and that the measurements of the right auricle are greater than those of the left in both males and females within the normal group. The observed differences between men and women may be attributed to the fact that auricular growth commences earlier in men than in women, continuing throughout their lifespan. No previous studies have performed anthropometric measurements in diabetic patients; however, if our findings of diabetics with type 2 are compared to the normal patients, all parameters were decreased, but with no major differences, except for the total ear width (TEW) similar findings were sen as per study done by Al-Saaeed in Iraq [7]. As per study done by Rani *et al.* the mean value of all the indices in the case of both males and females was higher in the left ear only except for the auricular index of males [8]. All of these modifications may hold significance due to the connective tissue over an extended period. Alterations in the composition and amount of the extracellular matrix's structural macromolecules affect the vessels and tissues. Diabetes mellitus is linked to various macro- and micro-molecular complications, along with neuropathic changes that can lead to hearing impairment, with the risk being heightened in individuals with diabetes compared to those without, primarily due to insufficient glycemic control.

## Conclusion:

There are significant anthropometric differences in external ear morphology between diabetic and normal individuals. This is attributed to chronic hyperglycemia-induced changes in connective tissue structure, including non-enzymatic glycation of collagen and micro vascular complications that affect skin elasticity and thickness.

## Sponsorship:

Nil
